# Drug therapy problems and contributing factors among patients with epilepsy

**DOI:** 10.1371/journal.pone.0299968

**Published:** 2024-03-07

**Authors:** Yirga Legesse Niriayo, Tesfay Gebregziabher, Gebre Teklemariam Demoz, Nigusse Tesfay, Kidu Gidey

**Affiliations:** 1 Department of Clinical Pharmacy, School of Pharmacy, College of Health Sciences, Mekelle University, Mekelle, Tigray, Ethiopia; 2 Clinical Pharmacy and Pharmacy Practice Unit, Departments of Pharmacy, College of Health Sciences, Aksum University, Axum, Tigray, Ethiopia; 3 School of Dental Medicine, College of Health Sciences, Mekelle University, Mekelle, Tigray, Ethiopia; Kaohsuing Medical University Hospital, TAIWAN

## Abstract

**Background:**

Although antiseizure medications play a crucial role in the management of epilepsy, their benefit can be compromised due to drug-related problems. Drug therapy problems can lead to poor seizure control, reduced quality of life, and increased morbidity and mortality in patients with epilepsy. However, in our setting, there is limited knowledge about drug therapy problems and the factors that contribute to them.

**Objective:**

The aim of this study was to investigate the prevalence and contributing factors of drug-therapy problems among patients with epilepsy.

**Methodology:**

A hospital-based prospective observational study was conducted at the neurologic clinic of Ayder Comprehensive Specialized Hospital, located in the Tigray region of Northern Ethiopia. The study included adult patients diagnosed with epilepsy who had been taking at least one antiseizure medication for a minimum of six months. Data were collected by conducting patient interviews and expert reviews of medical and medication records. Prior to data review and interviews, each patient provided written informed consent. Drug therapy problems were identified and classified using Cipolle’s method, followed by a consensus review conducted with a panel of experts. Statistical analysis was performed using a statistical software package; SPSS version 22. Binary logistic regression analysis was conducted to determine the contributing factors of drug therapy problems. Statistical significance was determined at p<0.05.

**Results:**

A study conducted on 250 participants revealed that 55.2% of the patients experienced one or more drug therapy problems. Our analysis identified a total of 282 drug therapy problems, with a mean of 2±0.52 drug therapy problems per patient. The most commonly observed drug therapy problems were dosage too low (30.0%), noncompliance (22%), adverse drug reaction (18%), and unnecessary drug therapy (16.4%). The commonly involved antiseizure medications in these drug therapy problems were phenytoin (22.8%), Valproic acid (20.8%), and Phenobarbital (18.4%). Furthermore, our findings revealed that combination therapy (AOR: 3.92, 95%CI: 1.19–12.97) and uncontrolled seizure (AOR: 108.37, 95%CI: 38.7–303.6) exhibited significant associations with drug therapy problems.

**Conclusion:**

Drug therapy problems were prevalent among patients with epilepsy. The use of combination therapy and the presence of uncontrolled seizures were identified as significant indicators of drug therapy problems. Therefore, more emphasis should be given to patients with multiple medications and uncontrolled seizures.

## Introduction

Antiseizure medications are the mainstay of therapy in the management of epilepsy and are indicated for patients who have experienced two or more unprovoked seizures, or a single unprovoked seizure accompanied by additional factors that increase the risk of seizure recurrence [1, 2]. The ultimate goal in managing epilepsy is to achieve complete seizure control without inducing adverse effects [3].Therefore, treatment should be individualized and optimized to achieve better control of seizures while preventing or minimizing adverse drug- related harms and complications [2, 4].

The majority of people with epilepsy can become seizure-free with the optimal use of currently available antiseizure medications [2, 5]. However, seizure control remains suboptimal in the majority of patients with epilepsy in developing countries, including Ethiopia [6, 7]. Despite the availability of several evidence-based antiseizure medications, there is a huge gap in the treatment of epilepsy in resource-limited countries, including Ethiopia, due to poor healthcare system, low health literacy, insufficient supply of antiseizure medications, and poor beliefs about medication in modern medicine [8, 9]. Different cultural backgrounds influence the treatment of epilepsy by shaping beliefs, stigmas, and cultural practices, which in turn impact medical accessibility, treatment adherence, and social integration [10, 11]. In Ethiopia, cultural beliefs and practices often result in the use of traditional medicine, religious rituals, or misconceptions about epilepsy, which can hinder access to appropriate medical treatments and result in the stigmatization of individuals living with epilepsy [6, 8, 12, 13]. Additionally, resource-limited countries face challenges related to climate change, such as global warming, that can adversely affect the accessibility and availability of antiseizure medications and hinder seizure control [14].

Drug therapy problems (DTPs) are defined as any unwanted occurrences associated with medication therapy, which can either potentially or actually hinder the intended objectives of the treatment [15]. DTPs can be categorized into seven main types, including unnecessary drug therapy, the need for additional drug therapy, ineffective drug therapy, dosage too low, dosage too high, adverse drug reactions (ADRs), and non-compliance [15, 16]. Drug therapy problems pose substantial challenges to public health globally, leading to increased morbidity, reduced quality of life, increased health care costs, and in severe cases, even mortality if not promptly identified and addressed [17–19].

Optimal use of antiseizure medications has been associated with improved quality of life and reduced morbidity and mortality in patients with epilepsy [20, 21]. However, most patients with epilepsy are not receiving appropriate treatment in developing countries [11, 22]. Although antiseizure medications play an important role in the management of epilepsy, their benefit can be compromised by drug-related problems [23, 24]. DTPs have been associated with reduced quality of life, increased morbidity, and mortality in patients with epilepsy [25–27]. Various factors can contribute to DTPs, such as the total number of medications being taken, reluctance of clinicians to implement clinical practice guidelines recommendations, absence of well-defined protocols, comorbidity, and seizure frequency [28–30].

In our specific setting, there has been a lack of thorough investigation and documentation regarding antiseizure medications related DTPs. Hence, it is crucial to conduct a study that can help identify, quantify, and document these issues in patients with epilepsy. This study will not only elucidate the extent of these problems but also highlight the existing gap in healthcare practice. By doing so, it will draw the attention of healthcare professionals and policymakers towards minimizing and preventing the occurrence of these problems. Therefore, the objective of this study was to examine the prevalence and underlying factors associated with DTPs in the management patients with epilepsy.

## Methodods

### Study design and setting

A prospective observational study was conducted at the neurology clinic of Ayder Comprehensive Specialized Hospital (ACSH) in the Tigray region of Northern Ethiopia between January 2019 and April 2019. ACSH is a teaching and referral hospital that serves approximately 10 million people in the catchment area.

### Study participants

The study included adult patients (aged >18 years) diagnosed with epilepsy, who had been regularly followed up for at least six months and were taking at least one antiseizre medication. Patients with intellectual disabilities or serious illnesses that prevented them from completing the interview, those who did not provide consent, and those with incomplete medical records were excluded. We calculated a sample size of 250 patients using a single population proportion formula, assuming a 50% proportion of DTPs, a 5% margin of error, a 95% confidence level, and a 10% contingency for nonresponse rate. Patients were enrolled in the study through a random selection process during their medication refilling appointments, using a simple random sampling technique.

### Data collection instrument and procedure

A structured data collection tool, including a questionnaire and data abstraction checklist [19], was utilized to extract all necessary information. The questionnaire was translated into the local language, Tigrigna, and then back-translated into English to ensure consistency. The Tigrigna-language version questionnaire was evaluated for its content validity by a panel of three experts in the field, including two experts from the clinical pharmacy department and one expert from the internal medicine department. The Tigrigna-language version questionnaire exhibited good reliability with an internal consistent reliability (Cronbach’s α) coefficient of 0.84. A pre-test was conducted on 5% of the sample at a different hospital to assess face validity of the drafted questionnaire. Based on the results of this pre-test, minor amendments were made to the questionnaire to enhance clarity and eliminate any ambiguities.

All patients were followed up for a period of six months to detriment the occurrence of DTPs within this period. All participants provided written informed consent after receiving a detailed explanation of the study’s objectives. Data were gathered by conducting patient interviews and conducting expert reviews of medical, medication, and laboratory records of the patient. For this study, data collection was carried out by fifth-year clinical pharmacy students who received training on the study objectives and data collection methods.

### Drug therapy problems identification and assessment

We employed Cipolle’s method [15] to identify and classify drug therapy problems. Subsequently, we convened a consensus meeting with a panel of experts comprising medical neurologists and clinical pharmacist.

The appropriateness of antiseizure medications was evaluated based on the Ethiopian standard treatment guideline (STG), American Epilepsy Society (AES) and International Leagu Against Epilepsy (ILAE) [20, 31].

### Data analysis

The data were entered into Epi Info (version 7) and analyzed using the Statistical Package for the Social Sciences (SPSS version 22.0). All statistical tests were conducted using the SPSS software. Descriptive statistics were used to summarize the baseline characteristics of the patients, as well as the prevalence and type of DTPs. To examine multicollinearity among predictor variables, the variance inflation factor (VIF) was calculated, and no evidence of collinearity was found. To evaluate the association between each independent variable and DTPs, a univariate logistic regression analysis was conducted. Furthermore, independent variables that demonstrated p-values less than 0.25 in the univariate analysis were subjected to a multivariable binary logistic regression model to determine the predictors of DTPs. A statistical significance level of p-value < 0.05 was applied.

### Ethical approval and informed consent

Ethical approval for this study was obtained from the Ethics Review Committee of the School of Pharmacy, College of Health Sciences, Mekelle University. Each participant was fully informed about the purpose of the study, and written informed consent was obtained. All personal information was handled with utmost confidentiality and all procedures were carried out in strict compliance with approved institutional guidelines.

## Result

### Socio-demographic related characteristics

This study included a total of 250 participants. The average age (standard deviation (SD)) was 36.6 (15.5) years. Of the total sample, 60.8% were male, 62.8% resided in urban areas, and 45.2% were married. Approximately 30.8% of participants reported using traditional medicine for their illnesses. In terms of social drug use, 8.8% were alcohol consumers, 6.0% were smokers, and 3.2% were khat chewers (Table 1).

**Table 1:** Socio-demographic related characteristics among epileptic patients in ACSH, 2019 (n = 250).

Variable	Frequency (%)
**Sex**	
Female	98(39.2)
Male	152(60.8)
**Age**	
18–35	135(54.0)
36–60	94(37.6)
>60	21(8.4)
**Marital status**	
Single	102(40.8)
Married	113(45.2)
Divorced	31(12.4)
Widowed	4(1.6)
**Educational level**	
No formal education	73(29.2)
Primary education	88(35.2)
Secondary education	60(24.0)
College and above	29(11.6)
**Residence**	
Rural	93(37.2)
Urban	157(62.8)
Alcohol consumers	22(8.8)
Cigarette smokers	15(6.0)
Khat chewers	8(3.2)
Traditional medicine users	121(48.4)

### Disease and medication related characteristics

Approximately one-fourth (24%) of the patients had one or more comorbidities. The most common type of seizure was the generalized tonic-clonic seizure. The majority (54.8%) of the patients experienced seizures in the past six months, 54% were on combination therapy, and 52.4% had been on treatment for 5 years or more. The frequently used medications were phenytoin (43.2%), valproic acids (43.2%), and phenobarbital (38.4%) (Table 2).

**Table 2:** Disease related characteristics of epileptic patients in ACSH 2019 (n = 250).

Variable	Frequency (%)
**Comorbidities**	
Yes	60(24.0)
No	190(76.0)
Seizure type	
Generalized tonic clonic seizure	207(82.8)
Focal seizure	9(3.6)
Absence seizure	3(1.2)
Unclassified seizure	31(12.4)
**Seizure experience in the past 6 months**	
Yes	137(54.8)
No	113(45.2)
**Number of medications per patient**	
Monotherapy	118(47.2)
Combination therapy	132(52.8)
**Duration of treatment in a years**	
<5	131(52.4)
≥5	119(47.6)
**Commonly used medications**	
Phenytoin	108(43.2)
Valproic acid	108(43.2)
Phenobarbital	96(38.4)
Carbamazepine	59(23.6)

### Prevalence of drug therapy problems

We found one or more DTPs in 55.2% of the patients. A total of 282 DTPs with a mean of 2±0.52 DTPs per patient were identified. The most frequently identified DTP was dosage too low (30.0%), followed by noncompliance (22%), ADR (18%), and unnecessary drug therapy (16.4%) (Table 3).

**Table 3:** Types and causes of DTPs among epileptic patients in ACSH, 2019(n = 250).

Type of drug therapy problems	Causes of DTP	Proportions with regard to patients(n = 250), n (%)	Proportions with regard to DTPs (n = 282), n (%)
Unnecessary drugtherapy	Drug duplication	41(16.4)	49(17.2)
Needs additional drug therapy	Untreated indication	6(2.4)	6(2.1)
	Require additional pharmacotherapy to attain synergistic effect	3(1.2)	3(1.1)
Ineffective drug Therapy	Not the most effective among the available drugs	4(1.6)	4(1.4)
	Not effective for the condition	3(1.2)	3(1.1)
Dosage too low	Dose too low to produce the desired response	75(30)	103(36)
ADR	Undesirable reaction that was not dose-related	23(9.2)	27(9.45)
	Drug interaction caused an undesirable reaction	4(1.6)	4(1.4)
	Dosage regimen administered or changed too rapidly	16(6.4)	19(6.64)
	History of allergy of the prescribed drug	2(0.8)	2(0.7)
Dosage too high	Dose too high	2 (0.8)	2(0.7)
Non-adherence	The patient did not understand the instructions	12(4.8)	13(4.6)
	Unwillingness to take the drug	5(2)	5(1.75)
	Drug product was too expensive for the patient	22(8.8)	25(8.7)
	Drug product was not available for the patient	16(6.4)	17(5.9)

DTP, drug therapy problems, ADR, adverse drug reaction

### Drugs commonly involved in drug therapy problems

**Fig 1:**
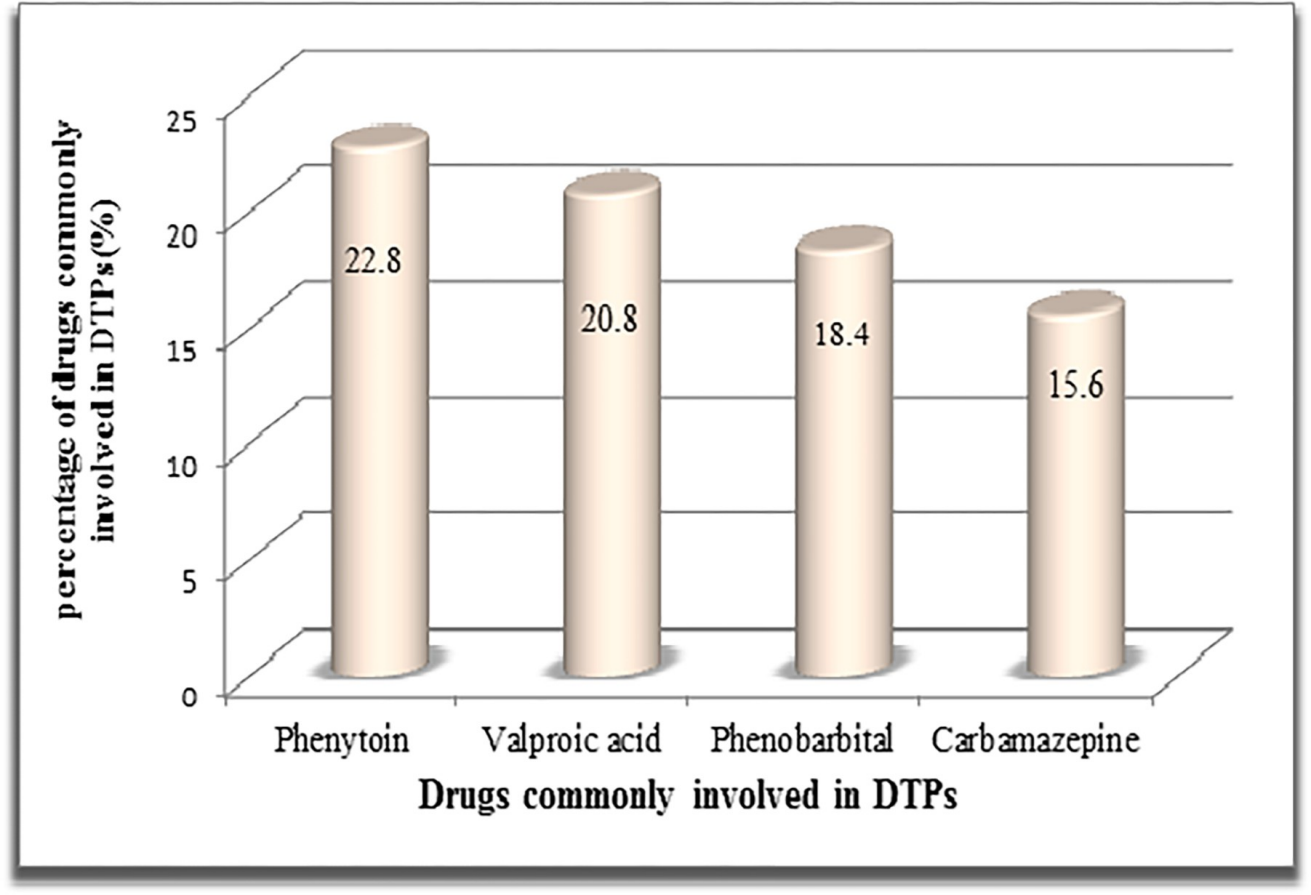
Drugs commonly involved in drug therapy problems in ACSH, 2019(n = 250).

Phenytoin (22.8%), valproic acid (20.8%), and Phenobarbital (18.4%) were the most commonly involved antseizure medications in DTPs (Fig 1). Valproic acid was often found to be associated with suboptimal dosage levels and cases of noncompliance. Additionally, insufficient dosage was commonly linked with carbamazepine and phenytoin, along with valproic acid. Adverse drug reactions (ADRs) were more frequently observed with phenobarbital and phenytoin. On a separate note, the most frequently duplicated drug treatments that often led to unnecessary drug therapy were a combination of phenytoin and phenobarbital, followed by carbamazepine and phenobarbital.

### Contributing factors to drug therapy problems

Factors contributing to drug therapy problems were examined through logistic regression analysis. In univariable logistic regression analysis, older age (COR: 3.22, 95%C: 1.18–8.80), residence (COR:1.713,95%CI:1.013–2.897), alcohol consumption (COR:9.32, 95%CI:2.13–40.81), uncontrolled seizure (COR:117.76, 95%CI:48.11–288.25), number of medications (COR:4.73, 95%CI:2.74–8.16), traditional medicine use (COR:1.79, 95%CI:1.027–3.12), and the presence of comorbidities (COR:3.13,95%CI:1.63–5.99) were significantly associated with presence of DTPs. Furthermore, independent variables that demonstrated p-values less than 0.25 in the univariate analysis were subjected to a multivariable binary logistic regression model to determine the predictors of DTPs. The overall model, including all predictors, yielded a statistically significant result (Chi-square = 217.832, df = 9, P<0.001). In the multivariate analysis, only number of medications (AOR: 3.92, 95%CI: 1.19–12.97) and uncontrolled seizure (AOR: 108.37, 95%CI: 38.7–303.6) remained significantly associated with DTP (Table 4).

**Table 4:** Factors associated with drug therapy problems.

Predictors		DTP		COR(95%CI)	AOR (95% CI)
		No, n (%)	Yes, n(%)		
Age category	18–35	76(56.3)	59(43.7)	(1)	(1)
	36–60	30(31.9)	64(68.1)	2.75(1.58–4.77)	2.80(0.97–8.09)
	>60	6(28.6)	15(71.4)	3.22(1.18–8.80)	0.87(0.174–4.33)
Number of medication	<2	83(61.5)	52(38.5)	(1)	(1)
	≥2	29(25.2)	86(74.8)	4.73(2.74–8.16)	3.92(1.19–12.97)
Seizure encounter	no	102(90.3)	11(9.7)	(1)	(1)
	Yes	10(7.3)	127(92.7)	117.76(48.11–288.25)	108.37(38.7–303.6)
Traditional medicine use	Yes	25(31.6)	54(68.4)	1.79(1.027–3.12)	1.603(0.55–4.62)
	No	87(50.9)	84(49.1)	(1)	(1)
Khat chewing	Yes	2(13.3)	13(86.7)	3.46(0.95–12.58)	1.98(0.17–20.79)
	No	110(46.8)	125(53.2)	(1)	(1)
Alcohol consumption	Yes	2(9.1)	20(90.9)	9.32(2.13–40.81)	4.27(0.27–67.53)
	No	110(48.2)	118(51.8)	(1)	(1)
Residence	rural	34(36.6)	59(63.4)	1.71(1.01–2.89)	1.06(0.37–3.02)
	Urban	78(49.7)	79(50.3)	(1)	(1)
Comorbidity	Yes	15(25.0)	45(75.0)	3.13(1.63–5.99)	1.48(0.37–5.85)
	No	97(51.1)	93(48.9)	(1)	(1)

**COR,** crude odds ratio**, AOD**, Adjusted odds, **CI**, confidence interval, DTP, drug therapy problems

## Discussion

Epilepsy management in Ethiopia faces significant challenges due to limited healthcare resources, a shortage of trained medical professionals, and societal stigma [8, 32, 33]. Access to specialized epilepsy services, including diagnostic tools, antiseizure medications, and adequately trained healthcare professionals, is limited, especially in rural areas [11]. The availability and affordability of antiseizure medications, which are crucial for managing epilepsy, often face challenges [34]. Additionally, misconceptions and cultural beliefs contribute to stigmatizing individuals with epilepsy, which hinders their acceptance and access to healthcare services [12, 13, 35]. As a result, epilepsy management in Ethiopia is suboptimal, leading to inadequate seizure control and negatively impacting the affected individuals’ quality of life [6, 11]. To address these challenges, it is imperative to invest in healthcare infrastructure, healthcare professional training, and public awareness campaigns to enhance epilepsy care in Ethiopia [36].

Despite the fact that majority of the individuals with epilepsy can become seizure-free with the optimal use of currently available antiseizure medications (2, 5), our study revealed that less than half (45.2%) of the patients attained seizure freedom during the study period. This may be attributed to the high prevalence of DTPs observed in our study.

Evidence-based guidelines recommend appropriate use of antiseizure medications to achieve seizure freedom in patients with epilepsy [20, 31]. However, more than half (55.2%) of the patients experienced DTPs in our study. In agreement with our study, DTPs were found in the majority of epileptic patients in in a study conducted in Malaysia [30]. The high prevalence of DTPs could be attributed to the absence of well-defined protocols, poor belief in modern medicine, poor health care system, and inadequate supply of antiseizure medications in developing countries, including Ethiopia [7–9, 37].

The frequently identified DTPs in the present study were dosage too low, noncompliance, adverse drug reactions (ADRs), and unnecessary drug therapy. Similar findings were also reported in Malaysia study [30]. Although evidence-based guidelines recommend intensification of antiseizure medications for optimal seizure control [20, 31], nearly one-third (30%) of the patients were taking low dose despite having uncontrolled seizure. Additionally, the study revealed that 22% of patients were non-adherent to their prescribed medications, despite the critical importance of adhering to antiseizure medications for effective patient care and treatment outcomes [38]. Consistent findings were reported in other similar studies [28, 39].

The most frequently implicated antiseizure medications in DTPs were Phenytoin (22.8%), valproic acid (20.8%), and Phenobarbital (18.4%). This observation could be attributed to the high prescription rates of these drugs in our study. It is important to note that drugs which are prescribed and used more often tend to have a higher probability of being associated with drug-related problems [40].

According to the present study, patients who received combination therapy were approximately four times more likely to experience DTPs compared to those on monotherapy. While monotherapy is generally recommended for the majority of epilepsy patients [41, 42], our study revealed that less than half (47.2%) of the patients were actually receiving monotherapy. This contrasts with previous studies, which have indicated a higher prevalence of monotherapy usage [43, 44]. This may be attributed to the lack of expertise among healthcare professionals and the absence of specific epilepsy treatment guideline in our setup [6].

Numerous studies demonstrated a negative association between drug therapy problems and optimal seizure control [3, 28, 45, 46]. Similarly, the majority of patients with uncontrolled seizure experienced drug therapy problems in our study. This finding indicates that additional measures should be taken to prevent and reduce DTPs in order to enhance the outcome of epilepsy treatment.

Finally, our study had the following limitations that should be acknowledged. Firstly, it is possible that patients may have underreported socially undesirable activities, such as non-adherence to medications. Additionally, our study excluded individuals with intellectual disabilities or severe illnesses that impeded their ability to participate in the interview, potentially biasing the representation of certain patient groups. While we attempted to explore various factors influencing DTPs, we did not assess the impact of healthcare professionals’ knowledge and qualifications, which could have significant implications. Our study did not directly employ diagnostic procedures to determine seizure types. Instead, the assessment of seizure types relied on the diagnoses documented by physicians during the course of patient care. Moreover, caution should be exercised when generalizing the findings of this study to other countries, as there may be variations in patient characteristics, disease distributions, healthcare systems, and research methodologies employed.

## Conclusion

Drug therapy problems were prevalent among patients with epilepsy. The most commonly identified DTPs included dosage too low, noncompliance with the prescribed medications, adverse drug reactions (ADRs), and unnecessary drug therapy. It was found that combining multiple medications and experiencing seizures were indicators of potential drug therapy problems. Consequently, it is imperative to prioritize patients who are on multiple medications and have uncontrolled seizures. Moreover, conducting longitudinal interventional studies would be beneficial in preventing and reducing the incidence of DTPs in epilepsy.

## Supporting information

S1 Checklist STROBE statement—checklist of items that should be included in reports of *cross-sectional studies*. (DOCX)

S1 Data (SAV)
